# Synthesis of CuO and Cu_3_N Nanoparticles in and on Hollow Silica Spheres

**DOI:** 10.1002/ejic.201201442

**Published:** 2013-03-08

**Authors:** Rupali Deshmukh, Ulrich Schubert

**Affiliations:** [a] Institute of Materials Chemistry, Vienna University of Technology1060 Vienna, Austria E-mail: Ulrich.Schubert@tuwien.ac.at Homepage: http://www.imc.tuwien.ac.at/

**Keywords:** Nanoparticles, Hollow spheres, Mesoporous materials, Copper, Nitridation reactions, Nitrides

## Abstract

Copper oxide nanoparticles within hollow mesoporous silica spheres were prepared by binding/adsorbing Cu^2+^ or [Cu(NH_3_)_4_(H_2_O)_2_]^2+^ ions on the surface of carbon spheres, followed by formation of a mesoporous silica shell by sol–gel processing and calcination in air. The CuO nanoparticles can subsequently be converted into Cu_3_N nanoparticles by nitridation with ammonia. The effect of the different copper precursors, i.e. Cu^2+^ and [Cu(NH_3_)_4_(H_2_O)_2_]^2+^, on the nanocomposites was studied. CuO nanoparticles on the outer surface of hollow silica spheres were obtained by thermal treatment of hollow CuSiO_3_ spheres in air. Nitridation of the CuSiO_3_ spheres with ammonia resulted in Cu_3_N@SiO_2_ composites, with aggregated Cu_3_N nanoparticles on hollow silica spheres.

## Introduction

Nanostructured inorganic materials with spherical hollow morphologies have received much attention because of their characteristic shape, well-controlled size, high surface area, large void space, and ready tailorability and functionalization of both the hollow cores and the shells.^1^,^2^ Such systems have many potential applications owing to their unique and interesting properties. Porous metal oxide capsules have the potential for interesting applications because the pores provide a diffusion path to and from the hollow cores. Among them, mesoporous silica shells are of great importance because of their high surface area, tunable pore size, chemical inertness, and thermal/mechanical stability.^3^ Porous hollow spheres with encapsulated metal or metal oxide nanoparticles can be used as nanoreactors.^4^–^6^

In this paper we describe the synthesis of CuO or Cu_3_N nanoparticles either within hollow mesoporous silica spheres or on their outer surface, in extension of our work on Cu_3_N nanoparticles embedded in a silica matrix.^7^ CuO–silica and Cu_3_N–silica composite nanostructures were recently investigated for various applications such as catalysis, photocatalysis, corrosion resistance, and optics.^8^ The chemical inertness, high temperature stability, and biocompatibility are important features of silica, which makes them ideal ingredients of composite materials. The incorporation of nanoparticles within or on hollow silica spheres provides structurally new functional nanomaterials.

## Results and Discussion

### CuO and Cu_3_N Nanoparticles in Hollow Silica Spheres

Hollow particles can be synthesized with the help of a sacrificial core template such as silica, carbon, or polystyrene spheres. Various types of metal oxide hollow spheres were obtained with carbon sphere templates.^9^ They are hydrophilic and inherently contain functional groups such as –OH, –CHO, –COOH on the surface, which can be used for binding metal ions without necessitating additional surface functionalization. After formation of the metal oxide shell, the carbon template is removed by thermal oxidation in air.

**Figure 1:**
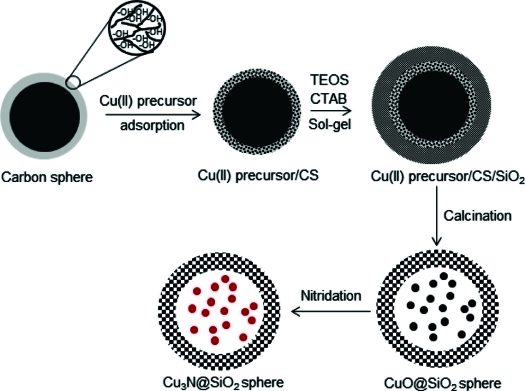
Schematic illustration of the preparation of Cu_3_N nanoparticles inside hollow mesoporous silica spheres.

The synthesis protocol for the rattle-type mesoporous Cu_3_N@SiO_2_ spheres described in this work is schematically shown in Figure 1 and consists of

1. adsorption of a copper(II) compound to the surface of 300 nm monodispersed carbon spheres (CS), which were prepared from an aqueous glucose solution according to Sun et al.,^10^

2. generation of a silica shell on the copper-loaded CS by sol–gel processing in the presence of a surfactant as porogen,

3. calcination of the Cu^II^/CS/silica composite spheres in air to remove the carbon core and the porogen, resulting in CuO nanoparticles within the hollow mesoporous silica spheres, and

4. conversion of the entrapped CuO nanoparticles to Cu_3_N nanoparticles by ammonia nitridation.

A similar approach (steps 1–3) was already reported for the synthesis of hollow porous silica spheres containing Pd^5^ or Fe_3_O_4_ nanoparticles.^6^

In the work presented here, Cu^2+^ or [Cu(NH_3_)_4_(H_2_O)_2_]^2+^ ions were adsorbed/bonded to the reactive functional groups on the CS particle surface. The copper-loaded carbon spheres were then coated with a silica layer by sol–gel processing of Si(OEt)_4_ (TEOS) in the presence of cetyltrimethylammonium bromide (CTAB). The surfactant has the role of a porogen; inter-particle porosity is created upon its thermal degradation. When the copper-loaded and silica-coated CS were heated in air, both the carbon core and the porogen were oxidatively removed. The (then porous) silica shell remained and CuO nanoparticles were formed within the hollow silica sphere.

**Figure 2:**
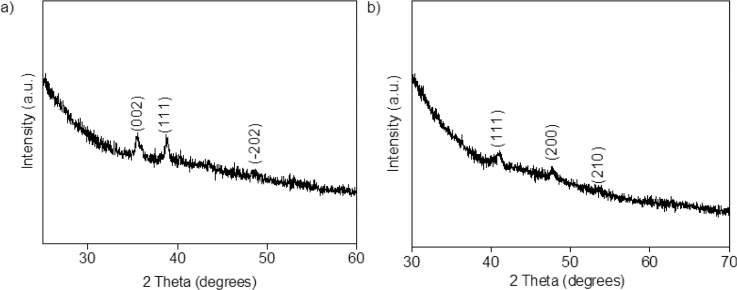
XRD patterns of CuO@SiO_2_(A) spheres (a) and Cu_3_N@SiO_2_(A) spheres (b).

Dispersion of the carbon spheres in an aqueous Cu(NO_3_)_2_ solution led to adsorption/binding of Cu^2+^ ions on the surface of the carbon spheres. TGA of the Cu^2+^-loaded and silica-coated CS [labeled Cu^2+^/CS/SiO_2_] (Figure S1) showed that the oxidation of the carbon spheres and organic constituents is essentially finished at 400 °C, with some char formation. Char is removed by further heating to ca. 470 °C. On the basis of the TGA analysis, the Cu^2+^/CS/SiO_2_ were calcined at 550 °C in air, and a black powder was obtained [labeled CuO@SiO_2_(A)]. The XRD pattern of CuO@SiO_2_(A) (Figure 2, a) revealed monoclinic CuO nanoparticles (JCPDS 41-0254). The broad hump at <30° is from the amorphous silica. The CuO crystallite size was 19 nm as calculated by Scherrer's equation. Nitridation of CuO@SiO_2_(A) at 300 °C under an ammonia atmosphere gave a brown product [labeled Cu_3_N@SiO_2_(A)], the XRD pattern of which corresponded to cubic Cu_3_N (JCPDS-86-2283, Figure 2, b). The average crystallite size of Cu_3_N was 17 nm, i.e. the crystallite size was preserved upon nitridation.

The nitrogen adsorption isotherms of CuO@SiO_2_(A) and Cu_3_N@SiO_2_(A) (Figure S2) are of type IV, which is characteristic of mesoporous materials, with some micro- and macroporosity (Table 1). CuO@SiO_2_(A) spheres have a high surface area, and the pore size distribution is centered at 2.5 nm. The surface area and pore volume are slightly lower for Cu_3_N@SiO_2_(A). This may be because of the basic conditions during nitridation, which might lead to restructuring of some mesopores/micropores.

**Table 1:** Surface and pore characteristics

Sample	Specific surface area [m^2^/g]	Pore diameter [nm]	Pore volume [cm/g]
CuO@SiO_2_(A)	953	2.5	0.78
Cu_3_N@SiO_2_(A)	671	2.3	0.53
CuO@SiO_2_(B)	747	2.6	0.70
Cu_3_N@SiO_2_(B)	523	2.4	0.56

**Figure 3:**
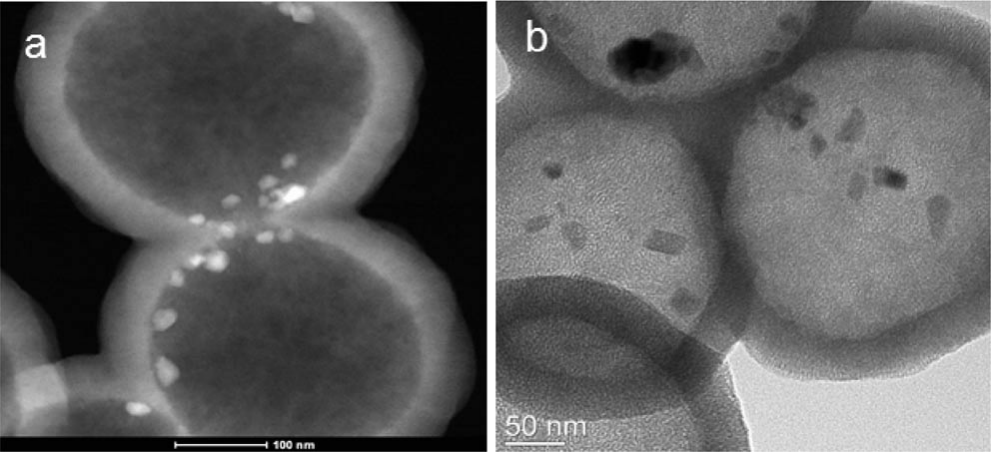
STEM (a) and TEM (b) images of Cu_3_N@SiO_2_(A) spheres.

The SEM image (Figure S3) of Cu_3_N@SiO_2_(A) shows that the spherical morphology is retained after calcination and nitridation. According to the EDX spectrum (Figure S4), the copper loading was 4 wt.-%. The STEM and TEM images of Cu_3_N@SiO_2_(A) spheres (Figure 3) show that the Cu_3_N nanoparticles are encapsulated in the hollow mesoporous SiO_2_ spheres. As seen in the STEM image, the Cu_3_N nanoparticles follow the shape of the sphere.

Although well-separated Cu_3_N nanoparticles with diameters <30 nm encapsulated in the hollow mesoporous silica spheres were obtained starting from Cu(NO_3_)_2_, the amount of Cu_3_N nanoparticles per silica sphere was limited. Therefore, the first step of the synthesis protocol, i.e. loading of the CS with a copper compound, was modified and the copper complex [Cu(NH_3_)_4_(H_2_O)_2_]^2+^ was employed instead of Cu(NO_3_)_2_. This modification is not just a change of the copper source, but also a change of the reaction conditions, because the employed aqueous [Cu(NH_3_)_4_(H_2_O)_2_]^2+^ solution is inherently basic. All subsequent steps were performed as described above.

TGA of [Cu(NH_3_)_4_(H_2_O)_2_]^2+^/CS/SiO_2_ (Figure S5) showed that oxidation of the organic constituents and the CS occurs at somewhat lower temperatures. For this reason, calcination of the composite in air was carried out at a lower temperature, i.e. 500 °C for 2 h. The obtained CuO nanoparticles in the hollow mesoporous SiO_2_ spheres [CuO@SiO_2_(B)] were converted to Cu_3_N nanoparticles [Cu_3_N@SiO_2_(B)] by nitridation at 350 °C for 2 h in ammonia. The crystallite sizes of CuO (22 nm) and Cu_3_N (15 nm) were in the same range as the particle sizes obtained by the first route.

The nitrogen adsorption isotherm of CuO@SiO_2_(B) and Cu_3_N@SiO_2_(B) (Figure S6) was again characteristic of mesoporous materials (type IV isotherm). The CuO@SiO_2_(B) spheres showed a surface area of 747 cm^3^/g, and the pore size distribution showed a sharp maximum centered at 2.6 nm. After nitridation the surface area was 523 cm^3^/g, and the pore size distribution was centered at 2.4 nm (Table 1).

**Figure 4:**
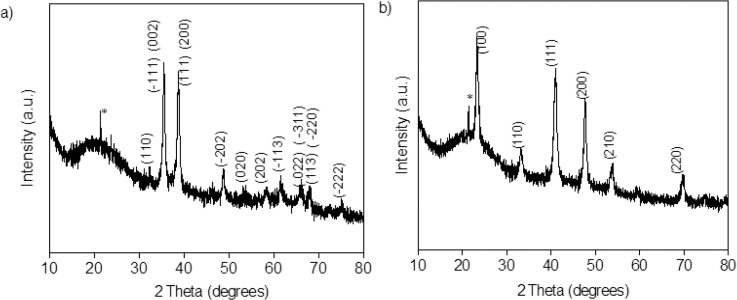
XRD pattern (* cristobalite) of CuO@SiO_2_(B) (a) and Cu_3_N@SiO_2_(B) (b).

The SEM image of CuO@SiO_2_(B) (Figure S7) showed that the spherical morphology of the template was also retained. Some broken spheres confirmed the hollow morphology. According to the EDX spectrum (Figure S8), the copper loading was 24 wt.-%, which is much higher than that for the particles obtained from Cu(NO_3_)_2_ as the copper precursor. The higher copper loading is also evidenced by the higher intensity of the reflections in the diffractograms of both CuO and Cu_3_N (Figure 4).

**Figure 5:**
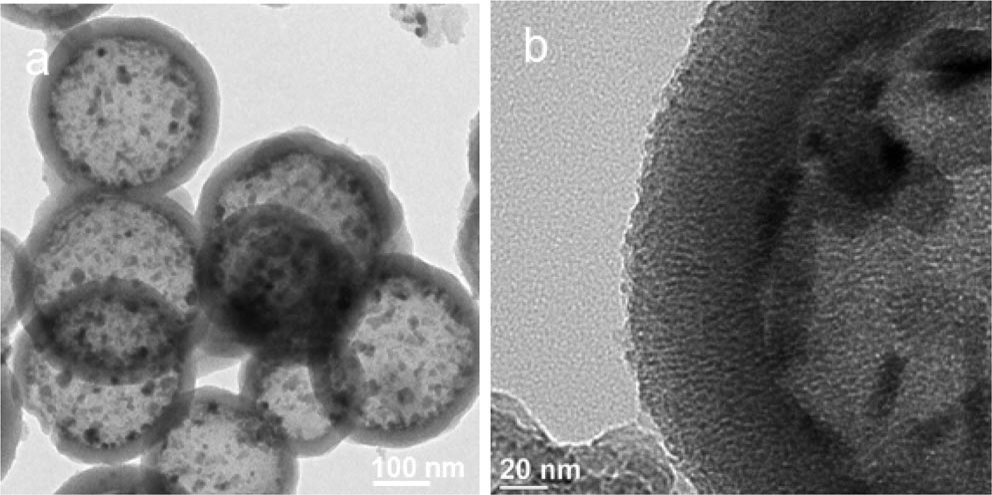
TEM images of CuO@SiO_2_(B) spheres.

**Figure 6:**
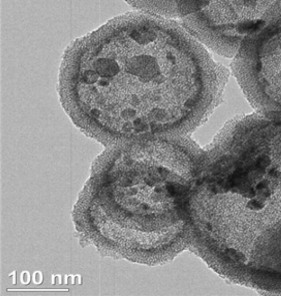
TEM image of Cu_3_N@SiO_2_(B) spheres.

TEM images of CuO@SiO_2_(B) (Figure 5) and Cu_3_N@SiO_2_(B) spheres (Figure 6) clearly confirmed that the CuO nanoparticles are exclusively located inside the hollow mesoporous silica spheres and that the morphology was not changed upon nitridation. The Cu_3_N particle size distribution appears to be broader than that of the CuO particles, or the Cu_3_N particles are more agglomerated/aggregated. The thickness of the silica shell is around 40 nm in the present case; it could easily be tuned by changing the weight ratio of the carbon sphere to TEOS.

### CuO and Cu_3_N Nanoparticles on Hollow Silica Spheres

The possibility of chemically converting metal silicate nanostructures into new chemical entities offers many options for the synthesis of new functional materials. For example, Jin et al. reported the synthesis of nickel-hollow silica sphere composites by hydrogen reduction of Ni_3_Si_2_O_5_(OH)_4_ at elevated temperatures. The hollow nanospheres exhibited high catalytic activity and good selectivity in acetone hydrogenation reactions.^11^

**Figure 7:**
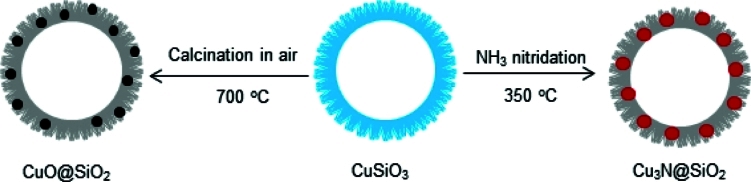
Synthesis of CuO on SiO_2_ and Cu_3_N on SiO_2_ from CuSiO_3_.

Hydrothermal treatment of Stöber silica spheres^12^ with copper nitrate under alkaline conditions at 140 °C results in the formation of hollow CuSiO_3_ spheres as reported by Wang et al.^13^ Synthesis of CuO and Cu_3_N nanoparticles on the surface of hollow silica spheres (labeled “CuO on SiO_2_” and “Cu_3_N on SiO_2_”) from the CuSiO_3_ spheres is schematically shown in Figure 7. Heat treatment in air at 700 °C led to the formation of CuO nanoparticles spread on the surface of the hollow silica spheres. Cu_3_N nanoparticles on the hollow SiO_2_ spheres were obtained by reaction of the CuSiO_3_ spheres with ammonia at 350 °C.

**Figure 8:**
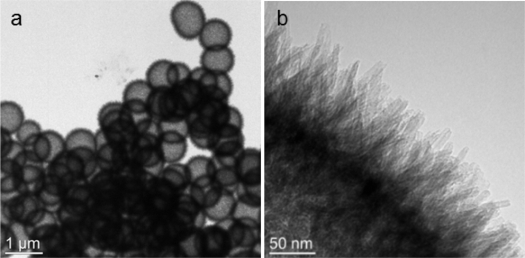
TEM images of the hollow CuSiO_3_ spheres.

Figure 8 shows TEM images of the starting CuSiO_3_ hollow spheres, which are composed of nanocrystals (Figure S9). Special features are nanotubes (Figure 8, b), which are arranged vertically on the surface of the spheres and were assumed to also consist of CuSiO_3_.^13^ Similar morphologies were also observed for other hollow spheres obtained by leaching processes.^14^

**Figure 9:**
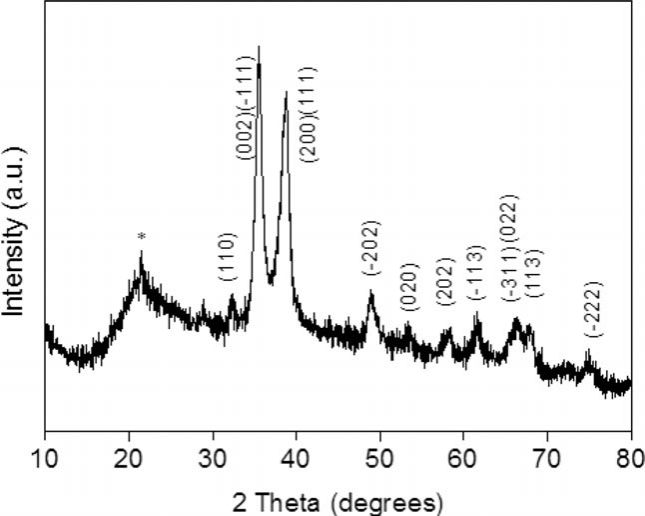
XRD pattern of CuO on SiO_2_ (* cristobalite).

Heat treatment of the blue hollow CuSiO_3_ spheres at 700 °C in air led to the formation of the black CuO on the SiO_2_ composite through disintegration of the copper silicate. Temperatures below 700 °C did not result in the formation of CuO nanoparticles. The XRD pattern is clear evidence for the formation of monoclinic CuO nanocrystals (Figure 9). The crystallite size of the CuO crystals was 10 nm. The surface area and average pore size of CuO on SiO_2_ was 181 m^2^/g and 3 nm, respectively (Figure S10). The much lower surface area, compared with CuO@SiO_2_(A) or CuO@SiO_2_(B) (Table 1), is due to the different preparation method of the hollow silica spheres, where no template for mesopores, i.e. no surfactant, was involved.

**Figure 10:**
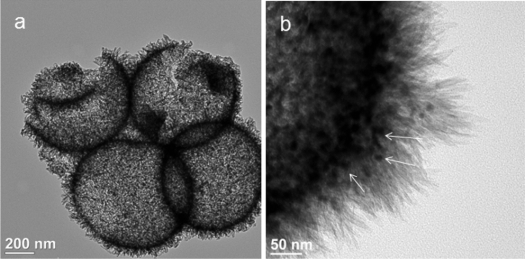
TEM images of CuO on SiO_2_. CuO nanoparticles are marked by arrows.

TEM (Figure 10) images clearly show that the morphology of the CuO on SiO_2_ composite is almost the same as that of the starting CuSiO_3_. The high magnification TEM image revealed that CuO nanoparticles are spread on the shell of the hollow silica spheres.

**Figure 11:**
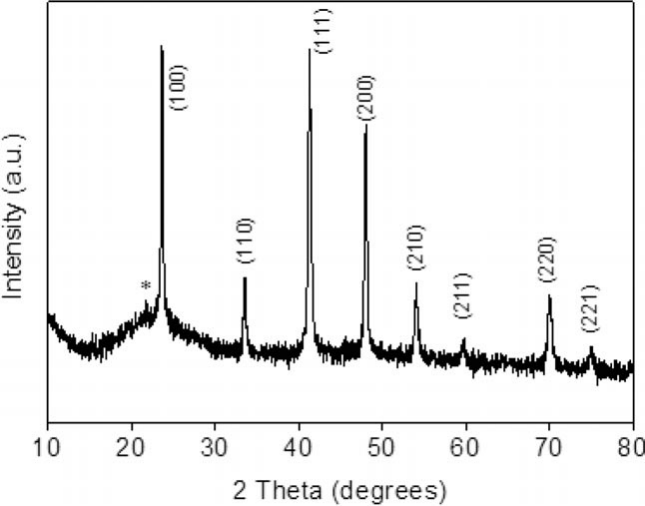
XRD pattern of Cu_3_N on SiO_2_ (* cristobalite).

After the nitridation reaction of the hollow CuSiO_3_ spheres with ammonia at 350 °C for 1 h, hollow silica spheres with Cu_3_N nanoparticles on the surface were produced without losing the hollow morphology of the CuSiO_3_ precursor. The nanotubes on the surface of the spheres were again retained. XRD analysis of the brown product (Figure 11) showed the presence of cubic Cu_3_N nanocrystals and amorphous silica as well as complete conversion from CuSiO_3_ to Cu_3_N on SiO_2_. The crystallite size of Cu_3_N crystals was 31 nm. The BET surface area was 159 m^2^/g and the pore size distribution from the adsorption branch of the isotherm was centered at 3–4 nm (Figure S11).

**Figure 12:**
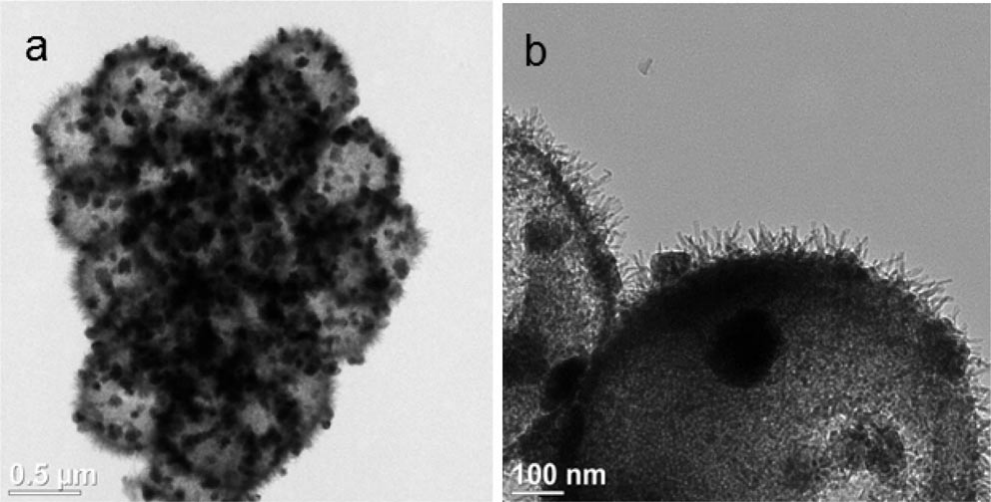
TEM images of Cu_3_N on SiO_2_.

SEM (Figure S12) and TEM analysis (Figure 12) of the Cu_3_N on SiO_2_ clearly showed Cu_3_N nanoparticles on the surface of the hollow silica particles.

Both reactions showed that the copper ions of the copper silicate structure migrated to the surface of the silica spheres during heat treatment or nitridation to form CuO and Cu_3_N nanoparticles, respectively. New morphologies of CuO on SiO_2_ and Cu_3_N on SiO_2_ composites were thus synthesized from the CuSiO_3_ precursor. Although the temperature during nitridation was much lower than during thermal decomposition, the obtained Cu_3_N nanoparticles were much larger than the CuO nanoparticles. Extraction of the Cu ions from the silicate network is apparently strongly favored when supported by a chemical reaction. Although a Cu_3_N nanoparticle size of 31 nm was determined from the XRD experiment, the particles appear to be much larger in the TEM micrographs. This indicates that the larger particles observed by TEM are in fact composed of smaller nanoparticles.

An interesting side aspect is that the nanotubular structures on the surface of the hollow particles were preserved. The XRD pattern after thermal decomposition (Figure 9) or nitridation (Figure 11) did not indicate any residual CuSiO_3_ phase. The investigation of the composition of the nanotubular structures in the CuO on the SiO_2_ composites by electron microscopy failed because the nanotubes were easily deformed by the electron beam during the analysis. Although the chemical composition of the nanotubes could not be determined experimentally, the only reasonable possibility is that they consist of silica. One possibility is that the nanotubes originally consisted of CuSiO_3_, as postulated by Wang et al.^13^ and the copper ions were leached during the subsequent reactions. Another possibility is that the nanotubes consisted of pure silica from the very beginning.

## Conclusions

A multistep protocol for the synthesis of CuO or Cu_3_N nanoparticles within hollow mesoporous silica spheres was described, starting from carbon spheres as templates to which copper(II) precursors were adsorbed/bonded. This approach, which was previously proven successful for metal or metal oxide nanoparticles,^5^,^6^ was now extended to metal nitride nanoparticles. It is anticipated that this post-synthesis modification of metal oxide particles inside the hollow albeit porous capsule can be generalized to prepare other functional nanoparticles, such as other metal nitrides or chalcogenides, within hollow mesoporous silica spheres.

Variation of the copper precursor, i.e. Cu(NO_3_)_2_ vs. [Cu(NH_3_)_4_(H_2_O)_2_]^2+^ (and/or the concomitant different reaction conditions) was found to affect the loading of the carbon spheres and thus the amount of Cu_3_N nanoparticles per silica sphere. This allows controlling the Cu_3_N proportion in the final materials. Cu(NO_3_)_2_ and [Cu(NH_3_)_4_(H_2_O)_2_]^2+^ interact differently with the functional surface groups on the carbon spheres or the nature of these groups is changed by the more basic reaction conditions in the latter case.

The conversion of CuSiO_3_ to CuO on SiO_2_ and Cu_3_N on SiO_2_ is a new observation. In each case the hollow sphere morphology of the starting CuSiO_3_ was well preserved. It is anticipated that this approach can also be applied to synthesize other metal oxide and metal nitride/silica hollow hybrid systems.

## Experimental Section

Glucose and cetyltrimethylammonium bromide (CTAB) were purchased from Sigma Aldrich, Cu(NO_3_)_2_**·**3H_2_O 99.5 % and ethanol from Merck, aqueous ammonia (28–30 wt.-%) from Baker, Si(OEt)_4_ (TEOS) from Fluka, and anhydrous ammonia gas (99.98 %) from Messer Austria GmbH. All Chemicals were used as received. The carbon spheres were prepared from an aqueous solution of glucose under hydrothermal conditions at 180 °C according to the procedure reported by Sun et al.^10^

**Materials Characterization:** Thermogravimetric analysis (TGA) was performed with a Netzsch TG 209C Iris at a heating rate of 10 °C/min under synthetic air. X-ray powder diffraction (XRD) measurements were performed with a PANalytical X′Pert PRO Bragg–Brentano X-ray powder diffractometer using Cu-*K*_α1_ radiation (*λ* = 1.5406 Å). Scanning electron microscope (SEM) images and energy dispersive X-ray analysis (EDX) were obtained using a FEI QUANTA-200 SEM and transmission electron microscopy (TEM) as well as scanning transmission electron microscopy (STEM) images using a TECNAI F20-S-TWIN with field emission source operating at 200 kV. The powders were deposited on a carbon grid for TEM analysis.

Nitrogen sorption measurements at 77 K were carried out with a Micromeritics ASAP 2020. The samples were left standing in vacuo overnight at room temp. prior to measurement. The surface area was calculated according to Brunauer, Emmett, and Teller (BET) and the *t*-plot method, and the pore size distribution, pore diameters and pore volumes according to Barrett, Joyner, and Halenda (BJH) from the adsorption branch of the isotherm.

**Preparation of Cu_3_N@SiO_2_(A) Spheres:** Carbon spheres (200 mg) were dispersed in a Cu(NO_3_)_2_**·**3H_2_O solution (0.5 m, 50 mL) with ultra-sonication for 60 min, followed by gentle stirring for 15 h. The copper-loaded CS were washed several times with distilled water and separated by centrifugation and then dried at 80 °C for 6 h.

The copper-loaded CS (175 mg) were dispersed in water (25 mL) with ultrasonication for 30 min. CTAB (0.28 g) was dissolved in a mixture of water (29 mL) and ethanol (22.2 mL). Both solutions were then mixed with stirring, followed by addition of aq. ammonia (25 %, 0.9 mL). After stirring for 30 min TEOS (0.5 mL) was added dropwise. The reaction mixture was stirred for 60 h at room temp. The CS/TEOS weight ratio was kept at 2.7, and the molar ratio of TEOS/CTAB/NH_3_/EtOH/H_2_O was 1:0.34:5.3:168:1320. Cu^2+^/CS/SiO_2_ was obtained by centrifugation, followed by three lots of washing and dispersion in water and finally drying at 80 °C. Heating at 550 °C for 6 h in air gave CuO@SiO_2_(A) as a black powder.

The CuO@SiO_2_(A) spheres were nitridated at 300 °C for 10 h in a horizontal tube furnace, under ammonia atmosphere with a flow rate of 10 L/h. The furnace was allowed to cool in an ammonia atmosphere, and a brown product was obtained.

**Synthesis of Cu_3_N@SiO_2_(B) Spheres:** An aqueous ammonia solution was slowly added to 10 mL of a 0.5 m copper nitrate solution. Carbon spheres (100 mg) were dispersed into the dark blue solution with ultrasonication for 30 min followed by gentle stirring for 15 h. The CS loaded with [Cu(NH_3_)_4_(H_2_O)_2_]^2+^ were washed several times with distilled water and separated by centrifugation and then dried at 80 °C for 6 h.

The [Cu(NH_3_)_4_(H_2_O)_2_]^2+^-loaded CS (100 mg) were dispersed in water (10 mL) with ultrasonication for 30 min. CTAB (0.12 g) was dissolved in a mixture of water (13 mL) and ethanol (10 mL). Both solutions were mixed with stirring followed by addition of aq. ammonia (25 %, 0.38 mL). The mixture was stirred for 30 min, and finally 0.21 mL of TEOS was added dropwise. The reaction was stirred for 60 h at room temp. The weight ratio of [Cu(NH_3_)_4_(H_2_O)_2_]^2+^/CS to TEOS was 2. The molar ratio of TEOS/CTAB/NH_3_/EtOH/H_2_O was 1:0.34:5.3:168:1320. [Cu(NH_3_)_4_(H_2_O)_2_]^2+^/CS/SiO_2_ was obtained by centrifugation followed by three lots of washing and dispersion in water and finally drying at 80 °C. Heating at 500 °C for 2 h in air gave CuO@SiO_2_(B) as a black powder.

The CuO@SiO_2_(B) spheres were nitridated at 350 °C for 2 h under ammonia with a flow rate of 15 L/h. The furnace was allowed to cool in ammonia atmosphere, and a brown product was obtained.

**Synthesis of Stöber Particles:** A solution of 81 mL of ethanol and 24.5 mL of NH_3_ (28–30 %) was stirred with 750 rpm at 30 °C in a 500-mL round-bottomed flask sealed with a septum. TEOS (4.2 mL) was injected rapidly into this solution, and the reaction was stirred for 60 min. A colloidal suspension of silica spheres was obtained, which was centrifuged and washed with ethanol and water several times. The white solid was dried at 60 °C for 6 h.

**Synthesis of Hollow CuSiO_3_ Spheres:** In a typical experiment, SiO_2_ spheres (0.130 g) were dispersed in distilled water (30 mL) using ultrasonication. Cu(NO_3_)**·**3H_2_O (0.7 mmol) was separately dissolved in distilled water (30 mL) followed by addition of aq. ammonia (30 %, 3 mL). The homogeneous solution obtained after mixing the two solutions was transferred into an 80-mL Teflon-lined stainless steel autoclave. The autoclave was kept at 140 °C for 24 h in a preheated electric oven and was then allowed to cool to room temp. A blue product was isolated by centrifugation and was washed several times with water, followed by drying at 70 °C for 6 h.

**Synthesis of CuO on SiO_2_:** The hollow CuSiO_3_ spheres were placed in a ceramic boat. The boat was kept in a horizontal tube furnace at 700 °C for 30 min in air with a heating rate of 3 °C/min. Black CuO on SiO_2_ was obtained.

**Synthesis of Cu_3_N on SiO_2_:** The hollow CuSiO_3_ spheres were placed in a ceramic boat. The boat was kept in a horizontal tube furnace at 350 °C for 1 h in ammonia atmosphere with a heating rate of 3 °C/min. The NH_3_ flow rate was maintained at 10 L/h. Brown Cu_3_N on SiO_2_ was obtained.

**Supporting Information** (see footnote on the first page of this article): TGA analysis of Cu^2+^/CS/SiO_2_/ and [Cu(NH_3_)_4_(H_2_O)_2_]^2+^/CS/SiO_2_ composite spheres, N_2_ adsorption and desorption isotherms of CuO@SiO_2_(A), CuO@SiO_2_(B), Cu_3_N@SiO_2_(A), Cu_3_N@SiO_2_(B), CuO on SiO_2_, Cu_3_N on SiO_2_, SEM images of Cu_3_N@SiO_2_(A), CuO@SiO_2_(B), Cu_3_N on SiO_2_, EDX spectra of Cu_3_N@SiO_2_(A), Cu_3_N@SiO_2_(B), and XRD pattern of CuSiO_3_ spheres.
