# Supplementary material for: Synthesis of CuO and Cu3N Nanoparticles in and on Hollow Silica Spheres
Source: Eur J Inorg Chem. 2013 Mar 8;2013(14):2498–504. doi: 10.1002/ejic.201201442 (PMC3688256; doi:10.1002/ejic.201201442)
Supplement: Supplementary file 1 [file ejic2013-2498-SD1.pdf]

Eur. J. Inorg. Chem. **2013** · © WILEY-VCH Verlag GmbH & Co. KGaA, 69451 Weinheim, 2013 · ISSN 1099–0682

SUPPORTING INFORMATION

DOI: 10.1002/ejic.201201442
Title: Synthesis of CuO and Cu_3N Nanoparticles in and on Hollow Silica Spheres
Author(s): Rupali Deshmukh, Ulrich Schubert*

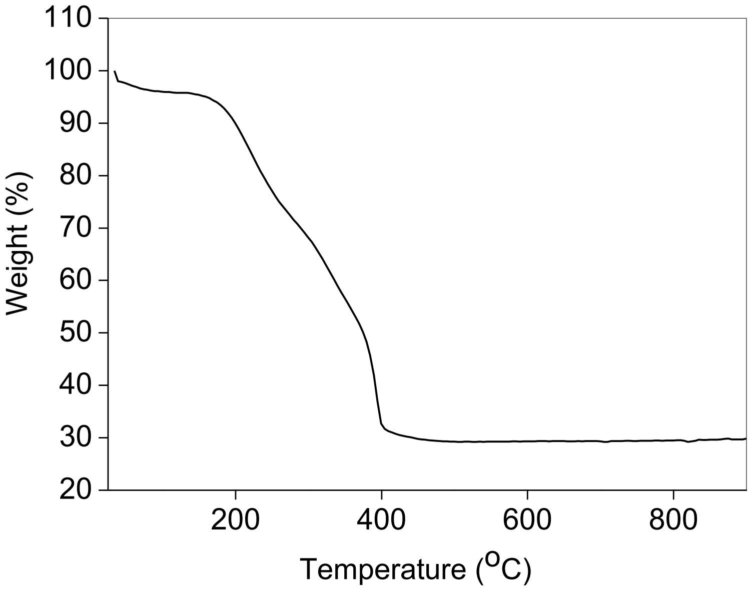

Figure S1 TGA of $Cu^{2+}/CS/SiO_2$ composite spheres.

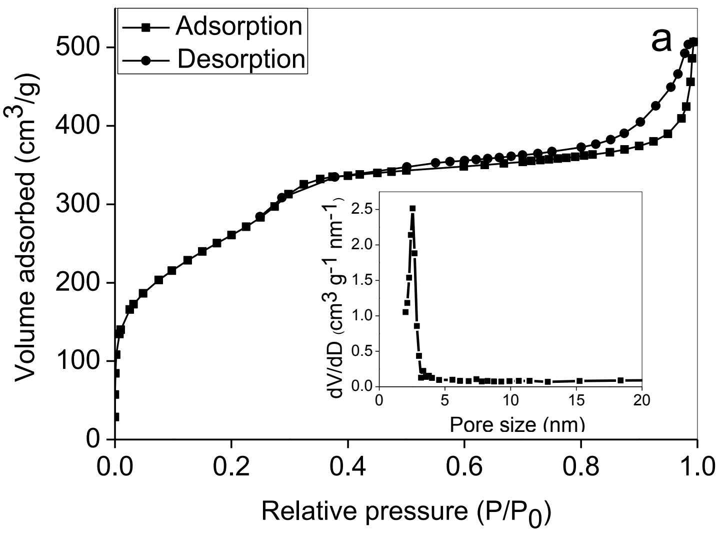

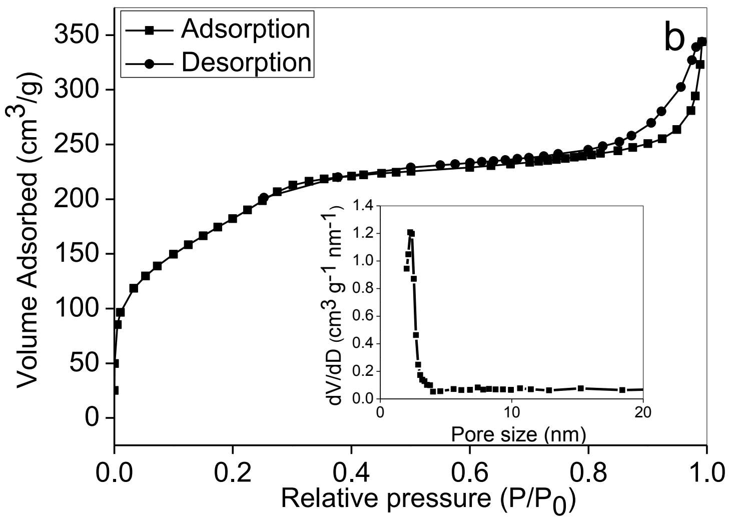

Figure S2 N_2 adsorption and desorption isotherms of (a) $CuO@SiO_2$(A) and (b) $Cu_3N@SiO_2$(A) (insets: pore size distribution for corresponding sample).

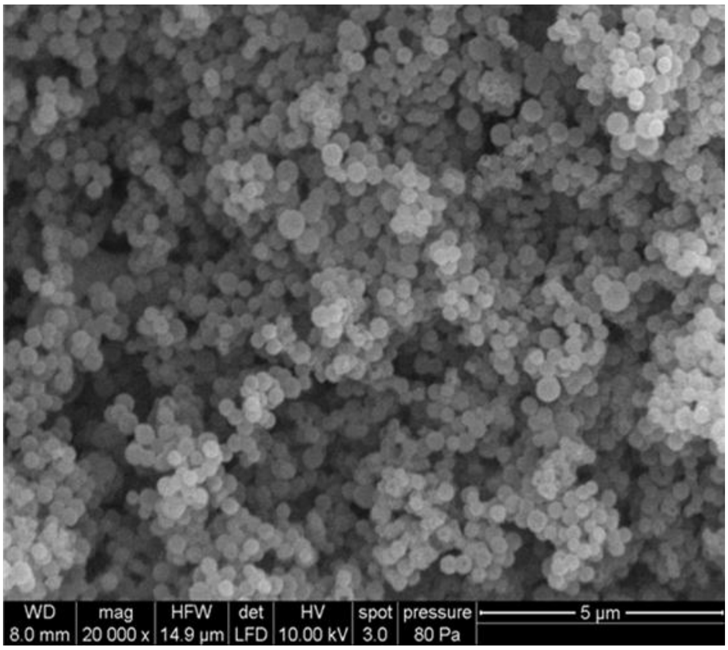

Figure S3 SEM image of $Cu_3N@SiO_2$(A) spheres.

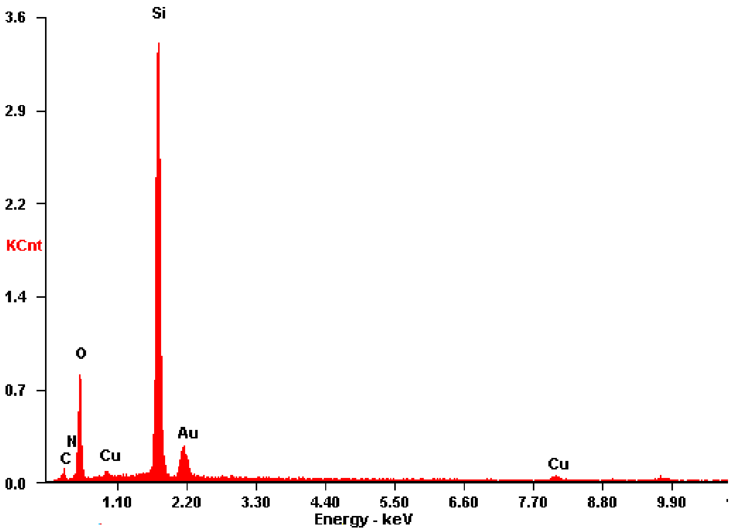

Figure S4 EDX spectrum of $Cu_3N@SiO_2$(A) spheres.

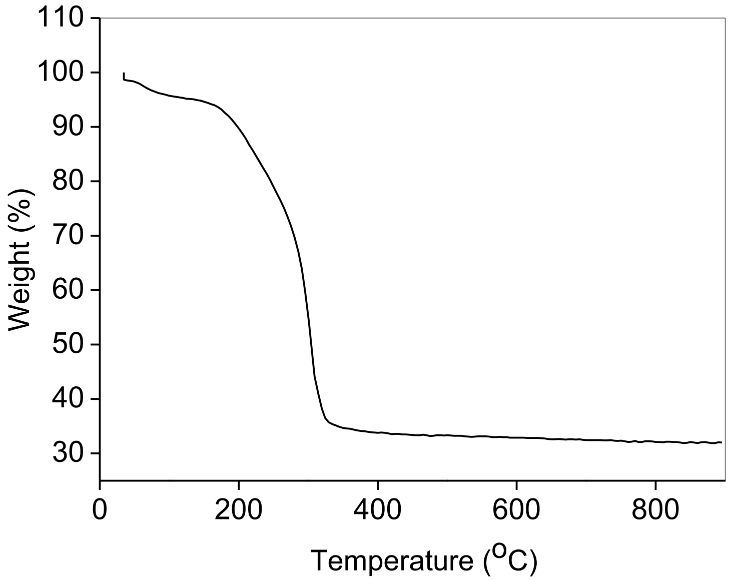

Figure S5 TGA of $[Cu(NH_3)_4(H_2O)_2]^{2+}/CS/SiO_2$ composite spheres.

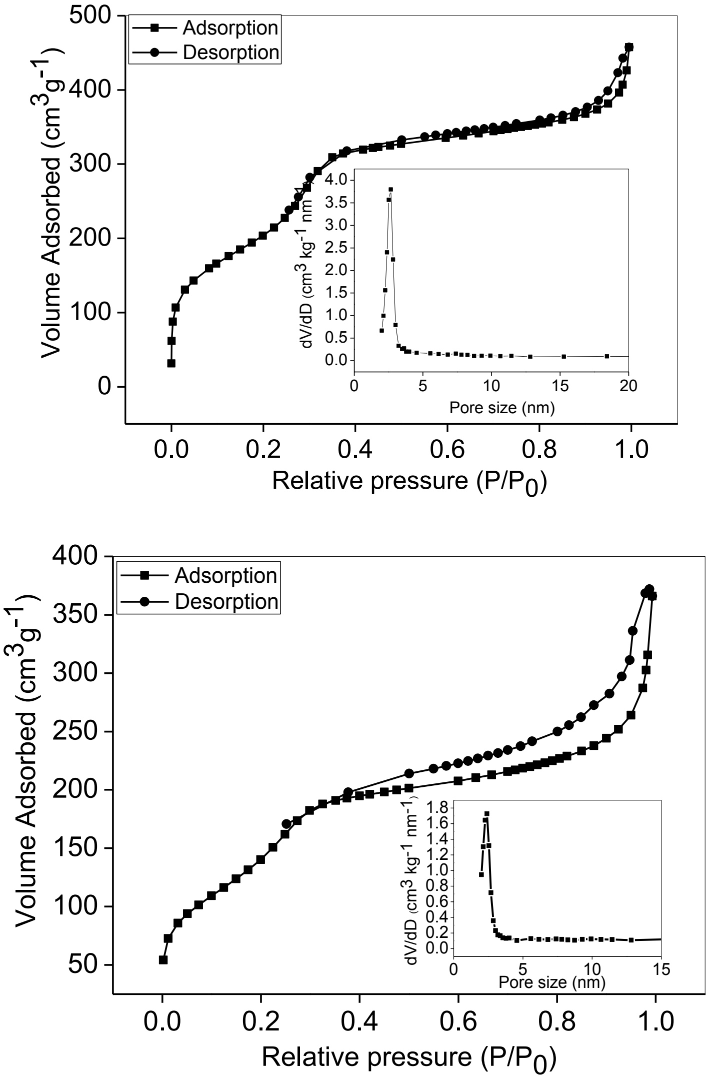

Figure S6 N_2 adsorption and desorption isotherms of CuO@SiO_2(B) (top) and Cu_3N@SiO_2(B) (bottom) (insets: pore size distribution for corresponding sample).

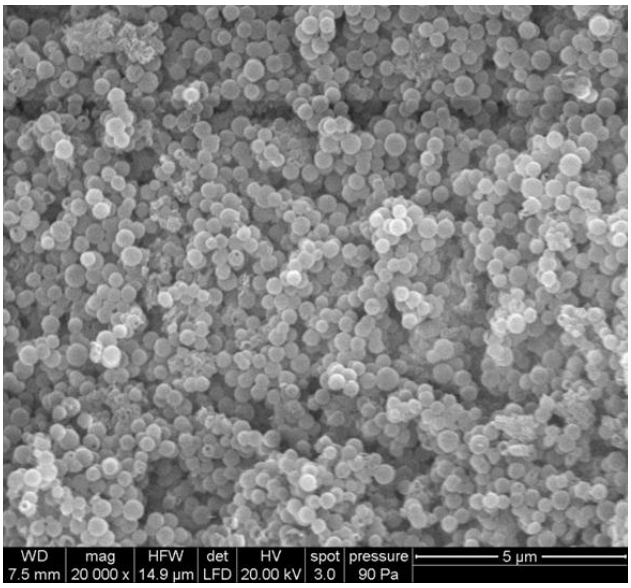

Figure S7 SEM image of $CuO@SiO_2$(B) spheres.

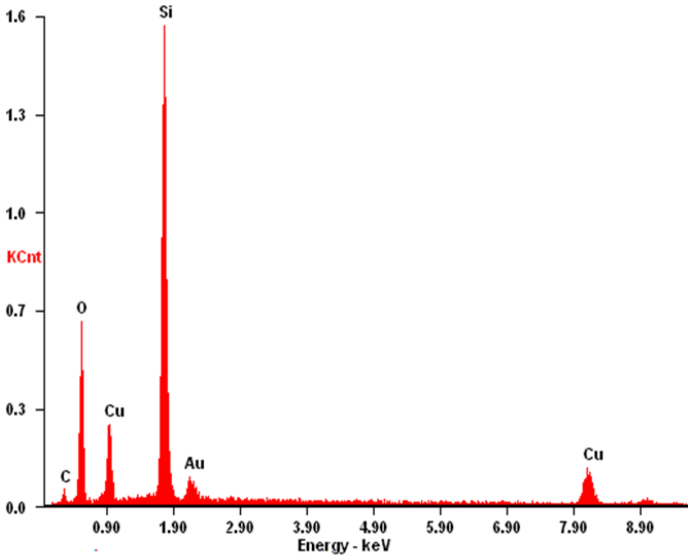

Figure S8 EDX spectrum of $CuO@SiO_2$(B) spheres.

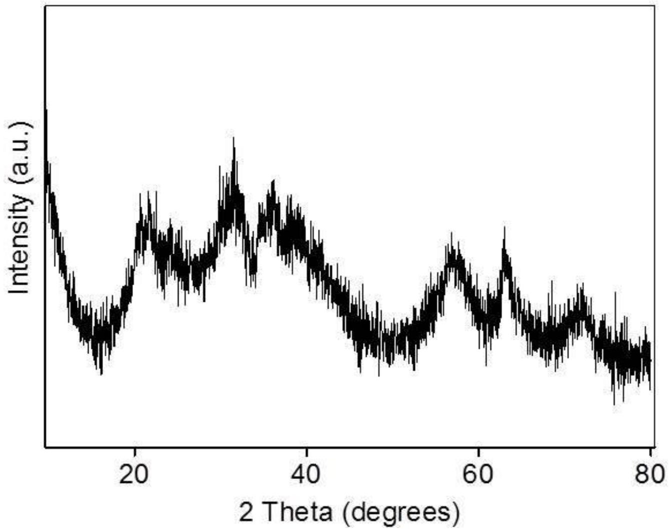

Figure S9 The XRD pattern of $CuSiO_3$ spheres (JCPDS 03-0219); the broadening of peaks is evidence that the material is nanocrystalline.

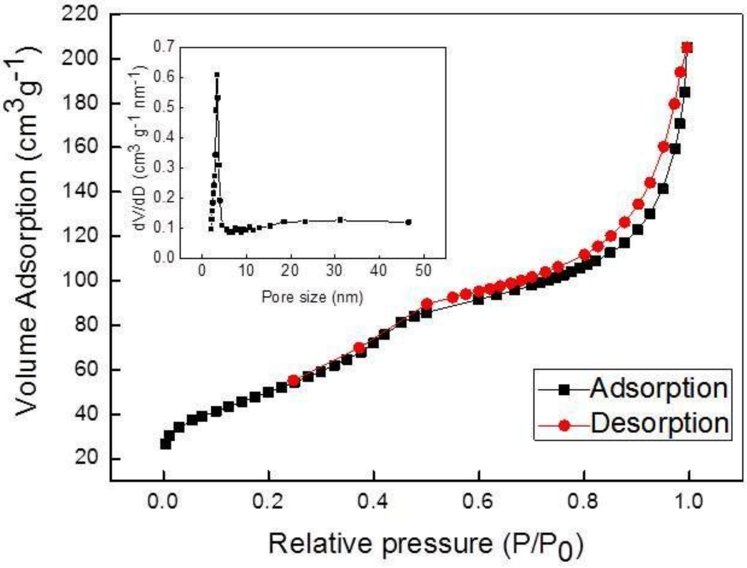

Figure S10 N_2 adsorption/desorption isotherm of CuO onSiO_2 (inset: pore size distribution from adsorption branch of isotherm).

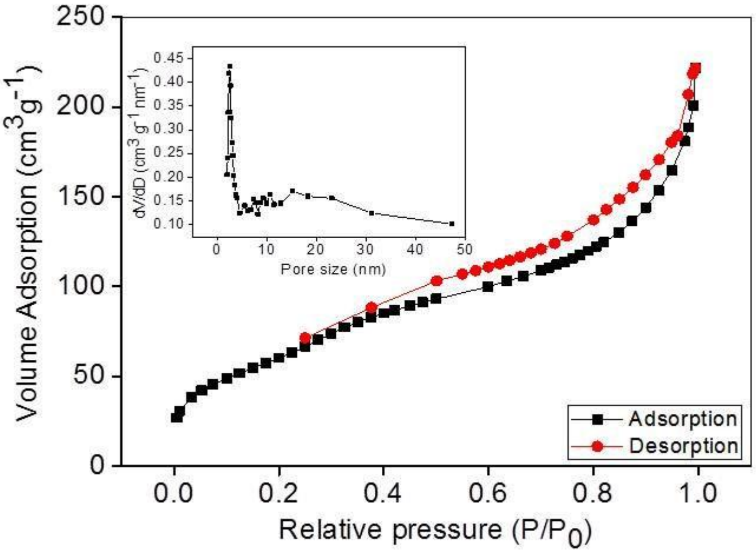

Figure S11 N_2 adsorption/desorption isotherm of Cu_3N on SiO_2 (inset: pore size distribution from adsorption branch of isotherm).

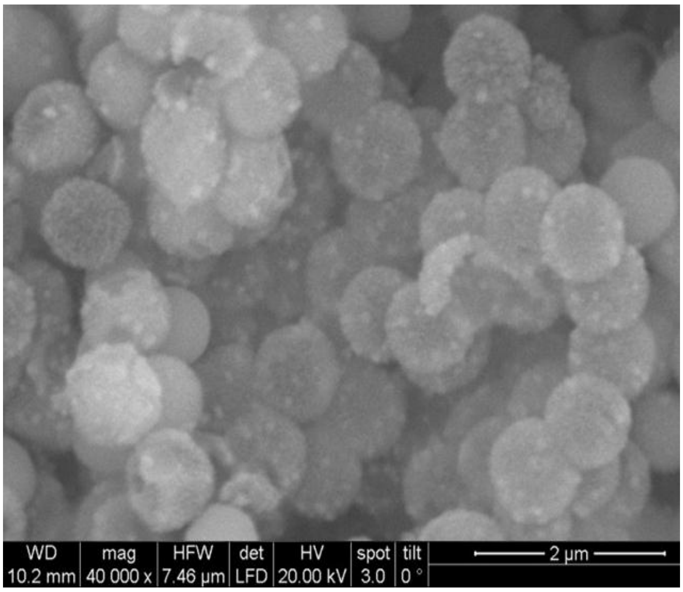

Figure S12 SEM image of Cu_3N on SiO_2. The white spots represent the Cu_3N nanoparticles.
